# Impact of Physical Activity and Sleep Duration on Depressive Symptoms in Hypertensive Patients: Results from a Nationally Representative Korean Sample

**DOI:** 10.3390/ijerph15122611

**Published:** 2018-11-22

**Authors:** Youn-Jung Son, Chanhee Park, Mi Hwa Won

**Affiliations:** 1 Red Cross College of Nursing, Chung-Ang University, Seoul 06974, Korea; yjson@cau.ac.kr (Y.-J.S.); kjaneditor@gmail.com (C.P.); 2 Department of Nursing, Wonkwang University, Iksan 54538, Korea

**Keywords:** hypertension, depressive symptoms, physical activity, sleep, adult

## Abstract

Depressive symptoms among individuals with hypertension may increase the risk of cardio-cerebrovascular disease, disease burden, and mortality. However, few studies have examined the relationships among physical activity, sleep duration, and depressive symptoms. Thus, this cross-sectional study examined the associations of physical activity and sleep duration with depressive symptoms in individuals with hypertension. We analyzed data collected as part of the 2014 Korea National Health and Nutrition Examination Survey, which included 846 patients with hypertension aged 19 or older. The prevalence rate of depressive symptoms was around 11.2%. A logistic regression analysis showed that moderate to vigorous physical activity (odds ratio (OR) = 4.42; 95% confidence interval (CI) = 2.19–8.89) and short (OR = 2.18; 95% CI = 1.11–4.28) and long sleep duration (OR = 4.09; 95% CI = 1.83–9.13) increased the risk of depressive symptoms after adjusting for confounding factors. Additionally, older age and low educational levels were associated with depressive symptoms. Our findings highlight that physical activity and sleep duration should be key components of lifestyle modification among hypertensive patients with depressive symptoms. Further investigation might benefit from validating these findings using a longitudinal design and examining the mediating effects of physical activity and/or sleep duration on the relationship between individual characteristics and depressive symptoms.

## 1. Introduction

Hypertension is a critical public health concern among aging populations worldwide because it increases the risk of cardio-cerebrovascular morbidity, such as myocardial infarction, heart failure, and stroke [1]. The prevalence rates of hypertension among the adult populations of England, the USA, and Canada are estimated to be 15–30% [2,3]. In 2015, hypertension was reported to affect approximately 28% of Korea’s adult population, of which 65% was aged 65 years and older [4]. Despite advances in antihypertensive treatment, poor control of hypertension remains a large financial burden on healthcare systems worldwide [1,2].

It is widely known that depressive symptoms are a risk factor for an increase in the incidence of uncontrolled hypertension as well as for the progression of cardiovascular disease [5,6]. A recent systematic review showed that approximately 27% of patients with hypertension experience depressive symptoms [7].

Depressive symptoms in hypertensive patients are associated with poor quality of life and health status as well as poor compliance to lifestyle modification therapy including physical inactivity, medication non-adherence, and increased mortality [6,7,8,9]. Some studies have identified that individuals with ischemic cerebrovascular changes owing to hypertension are more likely to develop depressive symptoms than the general population [8,10]. It is easier for patients with hypertension to experience depressive symptoms as a multidimensional construct or disorder consisting of affective, cognitive, behavioral, and somatic symptoms which have poor appetite, low energy, and insomnia [11,12]. Previous studies have shown that depressive symptoms are higher in individuals with hypertension compared to those without hypertension [8,9]. Accordingly, early recognition and appropriate management of depression in hypertensive patients has the potential to improve treatment adherence and prevent cardiovascular disease and stroke, thereby enhancing quality of life [5,13,14], For this reason, understanding the risk factors influencing depressive symptoms in hypertension patients is very important for healthcare professionals to reduce or treat depressive symptoms using a larger sample.

Regular physical activity has been shown to effectively decrease both depressive symptoms and high blood pressure (BP) [15,16]. Especially, regular moderate to vigorous physical activity can effectively help decrease depressive symptoms as well as prevent the development of depressive symptoms among patients with hypertension [17,18,19]. On the other hand, patients with depressive symptoms are less likely to engage in physical activity than those who are non-depressed [20,21]. Some studies reported that physical activity predicted depression in adults [22,23]. Unfortunately, there is a lack of information regarding the association between physical activity and depressive symptoms in Korean patients with hypertension.

Depressive symptoms are also associated with poor sleep quality including insomnia and abnormal sleep duration [24]. Many studies suggest that short and long sleep duration are associated with increased risks of morbidity and all-cause mortality [24,25,26]. A recent systematic review of patients with cardiovascular disease reported that abnormal sleep duration, whether too short or too long, increases the risk of depressive symptoms [26]. One longitudinal study found that insufficient sleep duration predicted risk of depressive symptoms [25]. Inappropriate sleep duration is also associated with low physical activity due to lack of energy and attention among patients with chronic disease [27]. In comparison with previous Korean data, recently, the proportion of adults who receive the appropriate amount of sleep—seven–eight hours per night—has been decreasing [28]. To date, few studies have explored the influence of physical activity and sleep duration, on depressive symptoms in patients with hypertension even though the possibility of bidirectional causality among these variables. In particular, despite the high rate of hypertension worldwide including in the Korean population, the link between these three factors among hypertensive individuals remains unclear. 

The Korea National Health and Nutrition Examination Survey (KNHANES) data resource is a nationwide cross-sectional survey conducted every year. KNHANES data are a valuable and representative source for monitoring changes in risk factors and diseases and identifying target groups in need of intervention. Based on the available evidence, we hypothesized that physical activity and sleep duration are associated with depressive symptoms in Korean patients with hypertension. Therefore, this study aimed to investigate the prevalence of depressive symptoms and the impact of physical activity and sleep duration on depressive symptoms in hypertensive patients after adjusting for individual characteristics using a nationally representative sample with the KNHANES data.

## 2. Materials and Methods

### 2.1. Setting and Sample

This cross-sectional study was based on raw data collected for the sixth KNHANES (2014), a nationally representative survey conducted by the Korea Centers for Disease Control and Prevention (KCDC). The KNHANES 2014, which was conducted to assess the health and nutritional status of the Korean population, used a stratified cluster sampling design based on geographic area, gender, and age group. Trained interviewers visited respondents’ homes and collected data through computer-aided face-to-face interviews from January to December 2014. The KNHANES 2014 survey involved 7550 participants out of 9701 respondents (participation rate: 77.8%). Of the 7550 participants, the data of 1149 participants aged 19 years or older who had been diagnosed with hypertension by a cardiologist were extracted. Data of 1088 participants who had systolic BP ≥ 140 mmHg or diastolic BP ≥ 90 mmHg and were taking antihypertensive medications were then extracted. Finally, after excluding respondents who had been diagnosed with stroke or cancer (*n* = 153) or had missing values on measures of marital status (*n* = 5), employment (*n* = 8), physical activity (*n* = 13), sleep duration (*n* = 19), or depressive symptoms (*n* = 44), data from 846 patients were included in the analyses.

### 2.2. Ethical Considerations

The data source for the KNHANES 2014 is publicly available and the participants’ information is recorded and provided by the KCDC in such a manner that they cannot be identified. Informed consent was obtained from all the participants for the KNHANES survey and the Ethics Committee Board of the KCDC approved the survey’s protocol (approval number: 2013-12EXP-03-5C).

### 2.3. Instruments

#### 2.3.1. Sociodemographic and Health-Related Characteristics

The patients’ sociodemographic characteristics included age, gender, residential area (urban or rural), educational level (below elementary school, middle school, high school, or college), marital status, employment, household income (quartiles), current smoking, and alcohol intake (monthly). Marital status was classified as not married (widowed, divorced, or single) or married. Household income was based on a standardized monthly income score (total monthly household income divided by the square root of the number of household members) and categorized into quartiles (low, low-middle, middle-high, and high income). Current smoking was classified as no (never a daily smoker, had smoked fewer than 100 cigarettes, or former smoker who had smoked at least 100 cigarettes in his/her lifetime, but did not smoke at the time of the survey) or yes (current smoker who had smoked at least 100 cigarettes in his/her lifetime and smoked on a daily or non-daily basis at the time of the survey). Alcohol intake was classified as no (less than once per month) or yes (once or more per month) based on drinking patterns during the previous month.

Health-related characteristics included comorbid conditions, time since hypertension diagnosis, daily medications, body mass index (BMI), systolic BP, and diastolic BP. Comorbid conditions were classified as “none”, “1”, and “2 or more” according to the number of chronic conditions participants suffered from at the time of the survey, including coronary artery disease (angina or myocardial infarction), diabetes, dyslipidemia, chronic renal failure, osteoporosis, and asthma. According to BMI, they were categorized as underweight (<18.5 kg/m^2^), normal (18.5–22.9 kg/m^2^), overweight (23–24.9 kg/m^2^), and obese (≥25 kg/m^2^) using the criteria for the Asia-Pacific region [29].

#### 2.3.2. Physical Activity

Physical activity was measured using the Global Physical Activity Questionnaire (GPAQ), which was developed by the World Health Organization (WHO) to assess the risk for chronic diseases. The GPAQ is a validated standardized questionnaire that is used worldwide. We used the Korean version of the GPAQ, which has been found to be a reliable and valid instrument [30]. 

The GPAQ assesses physical activity undertaken in 3 different domains: (1) work physical activity, (2) transport physical activity, and (3) leisure-time physical activity. The work and leisure-time domains assess the frequency and duration of 2 levels of intensity: vigorous intensity and moderate intensity. In the transport domain, only the frequency and duration of all walking and cycling for transport is evaluated. Total physical activity was estimated by summing the total metabolic equivalents (METs)-min of activities such as min/week spent in moderate and vigorous activities as well as the sum of both intensities (moderate intensity MET value = 4.0, vigorous intensity MET value = 8.0, and cycling and walking MET value = 4.0). One MET is defined as 1 kcal/kg/hour and is equal to the oxygen cost of sitting at rest. Therefore, when calculating a person’s overall energy expenditure using GPAQ data, 4 METs get assigned to the time spent in moderate activities, and 8 METs to the time spent in vigorous activities. Higher MET scores indicate higher levels of total physical activity.

According to WHO recommendations on global physical activity published in 2010 [31], throughout a week, including activity for work, during transport, and leisure time, adults between 18–64 or 65 years and above should do at least 150 min of moderate intensity physical activity, or 75 min of vigorous intensity physical activity, or an equivalent combination of moderate and vigorous intensity physical activity achieving at least 600 MET-minutes. 

Based on the WHO’s (2010) physical activity recommendations [31], subjects were classified into two categories: low physical activity (<600 MET-min/week) was considered “inactive” and moderate to vigorous physical activity (≥600 MET-min/week) was considered “active”.

#### 2.3.3. Sleep Duration

Sleep duration was assessed by a self-reported response to the following question: “How many hours do you sleep per night on average?” Sleep duration was classified into “short” (≤6 h/night), “normal” (7–8 h/night), and “long” (≥9 h/night) based on a previous report [25]. The duration of daytime naps was not taken into consideration in this study.

#### 2.3.4. Depressive Symptoms

Depressive symptoms were assessed using the Patient Health Questionnaire (PHQ-9), which was developed as a brief evaluation of mental disorders in primary care settings [32]. The Korean version of the PHQ-9 as a valid and reliable tool was adopted in this study [33]. The PHQ-9 is based on the Diagnostic and Statistical Manual of Mental Disorders IV (DSM-IV) criteria which provides a classification of mental disorders and includes nine items pertaining to depressive symptoms (e.g., “feeling down, depressed, or hopeless”), which are rated on a four-point scale from 0 to 3 in terms of their frequency (0 = not at all, 1 = several days, 2 = more than half the days, and 3 = nearly every day). The total possible score ranges from 0 to 27 points. Based on a previous study, when the PHQ-9 is used as a screening test, the most widely recommended cut-off value is 10, which has been suggested to indicate clinically significant depressive symptoms [32]. A meta-analysis study has shown that 17 validation studies concluding that the PHQ-9 has good psychometric properties (sensitivity 0.80, specificity 0.92) using the ≥10 cut-off score [34]. Namely, scores indicate severity of depressive symptom of the respondents (PHQ-9 < 10 = not clinically significant depressive symptoms vs PHQ-9 ≥ 10 = clinically significant depressive symptoms). Thus, we adopted the cut-off 10 which has been recommended as the cut-off for diagnosing this condition [32].

### 2.4. Statistical Analysis

The KNHANES is a nationwide study that uses stratified cluster sampling and weighted values. The present study analyzed the data using sample weights, stratification, and clustering, as recommended in the KNHANES data analysis guidelines [35]. Cross-sectional weights were used because the analysis was performed on data from only one year (i.e., 2014). All analyses used the sample weights from the KNHANES. Differences in characteristics between with and without depressive symptoms groups were compared using the Rao-Scott chi-square test or the independent *t*-test, and data were presented as either numbers or percentages.

Complex samples multiple logistic regression analysis was used to investigate the impact of physical activity and sleep duration on depressive symptoms after adjusting for sociodemographic and health-related characteristics applying stratified secondary weight respectively. To assess goodness of fit of the adjusted models, Nagelkerke R^2^ was also calculated. Before creating the logistic model, the independent variables significantly relevant to depressive symptoms were selected using bivariate analysis. Then, tolerance and the variance inflation factor (VIF) were examined to identify multicollinearity. According to Hair et al. [36], multicollinearity is a concern if the VIF is >5 and the tolerance is <0.20. The VIF values in this study ranged from 1.02 to 1.42 and the tolerance values ranged from 0.70 to 0.98. Thus, multicollinearity was not an issue. The results of the logistic regression model were presented as odds ratios (ORs) and 95% confidence intervals (CI). All estimates were calculated based on sample weights, which were evaluated by taking into consideration the stratified and cluster variables to generate the analysis-plan file. The analysis was adjusted for the complex sample design of the survey and performed using the statistical software SPSS version 23 (SPSS Inc., Chicago, IL, USA), with *p* < 0.05 as the level of statistical significance.

## 3. Results

### 3.1. Sample Characteristics and Prevalence of Depressive Symptoms

The mean (±SD) depressive symptoms score of the 846 participants was 3.58 (±4.74). The prevalence rate of depressive symptoms (PHQ ≥ 10) among individuals with hypertension was approximately 11.2% (*n* = 85). Table 1 depicts the statistically significant differences in age (*p* < 0.001), gender (*p* = 0.020), educational level (*p* < 0.001), marital status (*p* = 0.002), alcohol intake (*p* = 0.004), and comorbid conditions (*p* = 0.011) between the groups with and without depressive symptoms. However, there were no significant differences in residential area, employment, household income, current smoking, time since hypertension diagnosis, daily medication, BMI, mean systolic BP, or mean diastolic BP between those with and without depressive symptoms.

### 3.2. Differences in Physical Activity and Sleep Duration with and without Depressive Symptoms

In Table 2, the mean (±SD) physical activity scores of adults with and without depressive symptoms were 494.41 (±1116.89) and 1347.27 (±3941.09), respectively. In adults with depressive symptoms, the proportion of low physical activity (15.8%) was significantly higher than the proportion of moderate to vigorous physical activity (3.1%) compared to those who were not depressed (*p* < 0.001). 

With regard to sleep duration, the mean (±SD) nightly sleep duration scores of adults with and without depressive symptoms were 6.36 (±2.08) and 6.70 (±1.76), respectively. In adults with depressive symptoms, the proportions of short (10.8%) and long sleep duration (26.4%) were significantly higher than the proportion of normal sleep duration (4.6%) compared to those who were not depressed (*p* < 0.001).

### 3.3. Predictors of Depressive Symptoms in Adults with Hypertension

The logistic regression model revealed that low physical activity (OR = 4.42; 95% CI = 2.19–8.89), short (OR = 2.18; 95% CI = 1.11–4.28), and long sleep duration (OR = 4.09; 95% CI = 1.83–9.13) increased the risk of depressive symptoms after adjusting for sociodemographic and health-related variables, as shown in Table 3. 

In addition, older age (OR = 3.15; 95% CI = 1.37–7.24) and low educational level (elementary school or below) (OR = 3.06; 95% CI = 1.01–9.29) increased the risk of depressive symptoms in adults with hypertension, as shown in Table 3.

## 4. Discussion

Several studies have found that patients with depressive symptoms are more likely to be at higher risk for developing hypertension than are those without depressive symptoms, and that patients with comorbid hypertension and depressive symptoms are at a higher risk for cardio-cerebrovascular disease and all-cause mortality than those without these afflictions [7,10,13]. Meurs et al. [8] found that the prevalence rate of depressive symptoms in hypertensive patients is higher than in the general population. Thus, it is crucial to identify the risk factors for depressive symptoms in adults with hypertension. 

In the present study, the prevalence rate of depressive symptoms was about 11.2% based on a cut-off of PHQ ≥ 10. This result was relatively higher than in a study in the Netherlands wherein 5% of patients with hypertension had depressive symptoms using the same cut-off [37]. The Korea National Health and Nutrition Examination Survey that collected data from 2014 reported 6.7% prevalence of depressive symptoms (measured using the PHQ-9 ≥ 10) among Korean adults aged 19 and older (*n* = 4949) [38]. However, this was not consistent with the result of a recent systematic review of 41 studies [7]. This discrepancy might be owing to different assessment tools and cut-off scores for depressive symptoms in individuals with hypertension. Among many comparable measures for depression or depressive symptoms, the PHQ-9, as a self-reported measure, is considered reliable and is an internationally validated tool to assess clinical depression [32,39]. Nevertheless, the cut-off score for the screening of depressive symptoms in hypertensive patients can vary based on their cultural and socioeconomic backgrounds. Since 2004, Korea has seen the highest suicide rates among members of Organization for Economic Co-operation and Development (OECD) countries owing to depressive symptoms related to the extremely competitive and increasingly polarized Korean society [40]. In addition, despite their high prevalence and impact, depressive symptoms may be undiagnosed in patients with hypertension [39]. Accordingly, healthcare professionals should be aware of the importance of early and regular assessment for clinical depression among patients with hypertension. Furthermore, in the general population, it is vital to screen for and detect depressive symptoms before the development of high BP. A recent review found that early assessment of depressive symptoms in adolescence could be more important because adolescents are at elevated risk for the development of depression [41]. Thus, futures studies should focus on the efficacy of early identification and treatment of depressive symptoms on health conditions in healthy younger adults as well as older adults with hypertension.

Our main finding was that those who engage in low physical activity are at an increased 4.4 times higher probability of having depressive symptoms (PHQ-9 ≥ 10) than those who engage in moderate to vigorous physical activity. This result is in line with previous studies demonstrating that low physical activity predicts depressive symptoms in the adult population [15,39,40]. Win et al. [21] reported that low physical activity partially mediates the relationship between depression and mortality among persons diagnosed with cardiovascular disease. On the other hand, some studies have found that moderate to vigorous physical activity can improve mental well-being by reducing depressive symptoms [17,20]. Thus, healthcare professionals must encourage patients with hypertension to engage in regular physical activity of an appropriate intensity. According to the WHO guideline [31], forms of physical activity (PA) do not require specific skills or equipment and are easily accessible to most, even novices and currently inactive individuals. Lifestyle activities such as walking might be effective in managing hypertension [42,43], but patients and healthcare professionals are likely to ignore them. Particularly, South Korea has the highest business use of broadband internet access of the OECD countries, along with high levels of daily mobile use [44]. This is more likely to increase sedentary behaviors or physical inactivity. Accordingly, reducing the use of internet access or mobile phones may help increase PA among Korean adults regardless of comorbid hypertension and depressive symptoms. Further investigations are needed to identify what kind of exercise, including lifestyle activities, is effective in preventing or reducing depressive symptoms and to develop tailored physical activity guidelines according to the level of depressive symptoms among the population with hypertension. Furthermore, the social side of prevention, such as public health messages to increase PA in general populations as well as hypertensive patients, should take into account the opportunity available to individuals to perform altered activities into their lifestyle. 

In our logistic regression model, both short and long sleep duration also increased about 2.2 times and 4.1 times a higher probability of having depressive symptoms (PHQ ≥ 10) as compared to hypertension patients with normal sleep duration. This result was consistent with several studies regarding the relationship between short and long sleep duration and depressive symptoms [27,45,46]. Adequate sleep duration is vital for the regulation of body metabolism and maintaining quality of life [12]. Previous studies have shown that decreased sleep duration and quality are related to mental and physiological problems due to changes in the endocrine and immune systems [12,25,26]. In our study, the mean nightly sleep durations in adults with and without depressive symptoms were below and above seven hours, respectively. This finding provides further evidence of the fact that among the Korean adult population, the rate of appropriate sleep duration (seven–eight hours per night) has been decreasing [4]. Our finding implies the higher possibility of increased depressive symptoms among people with hypertension in Korea. However, more interestingly, long sleep duration in our sample predicted the higher risk of depressive symptoms compared to the impact of short sleep duration on depressive symptoms. Until now, studies on the association between long sleep duration and depressive symptoms in patients with hypertension have been relatively scarce compared to the studies regarding the association of short sleep with chronic diseases. Although sleep duration has been studied extensively in cardiovascular and other chronic disease, evidence regarding the relationship between sleep duration and depressive symptoms in hypertension is insufficient [25,27]. Accordingly, further investigation is necessary to explore the relationship between short or long sleep duration as well as other sleep patterns such as sleep quality, and daytime napping and the prevalence of depressive symptoms among patients with hypertension. Also, further prospective studies, with objective measures of sleep duration, are needed to examine the causal relation of sleep difficulties with depressive symptoms.

In the present study, we also found that uneducated older adults are more likely to have a 9 times higher risk of having depressive symptoms (PHQ ≥ 10) compared to those who are younger with higher educational levels. This finding supports the results of several studies that sociodemographic factors, including old age and lower educational levels, can increase the likelihood of developing depressive symptoms [27,46,47]. The elderly are often more likely to face significant life changes, such as disease, loneliness, and living alone as well as inadequate information about depressive symptoms, compared to younger populations [37,47]. In particular, older adults tend to confuse depressive symptoms such as fatigue and sleep problems with those of high BP [7]. Thus, it is necessary for healthcare professionals to consider age and educational background for lifestyle modification recommendations, including physical activity and sleep duration, in depressed patients with hypertension.

Our study has several methodological limitations. First, using a cross-sectional design with the KNHANES data precludes the ability to infer causal relationships. Second, the measurement of depressive symptoms and sleep duration was based on participants’ self-reports. Self-report measures are susceptible to inaccuracy because of social desirability and recall biases. Third, even though at the most frequently recommended cut-off point of 10, its sensitivity and specificity are high based on the PHQ-9 guideline, we had lower sensitivity (67%) and high specificity (93%) at the cut-off of 10 for detecting depression in our Korean sample. Thus, future studies are needed to use receiver operating characteristics (ROC) analysis to have acceptable screening accuracy for detecting major or clinical depression. Finally, this study could not examine potential biases because it used secondary data from the KNHANES 2014 survey. 

Nevertheless, to the best of our knowledge, this study is the first to identify the associations of physical activity and sleep duration with depressive symptoms in patients with hypertension using a relatively recent and reliable survey of a nationally representative sample of Koreans with hypertension. This study suggests that more research is needed to investigate causal relationships among physical activity, sleep duration, and depression in the general population, as well as patients with chronic diseases.

## 5. Conclusions

Our findings highlight that it is important to assess depressive symptoms early and identify the level of physical activity and sleep duration patterns in depressed adults with hypertension. Older age and low educational levels are also associated with depressive symptoms. Thus, healthcare professionals should consider individual backgrounds in the context of lifestyle modification for patients with depressive symptoms. Further studies are needed to examine the causal relationships among physical activity, sleep duration, and depressive symptoms using a larger cohort sample from diverse socioeconomic status and cultural backgrounds.

## Figures and Tables

**Table 1:** Comparison of characteristics of hypertension patients with and without depressive symptoms (*n* = 846).

Variables	Without Depressive Symptoms		With Depressive Symptoms		*p*-Value ^a^
	(*n* = 761, *N* = 3,835,776)		(*n* = 85, *N* = 379,127)		
	*n* or Mean ± SD	Weighted %	*n* or Mean ± SD	Weighted %	
Age (years)	65.87 ± 10.18		70.42 ± 8.69		
24–64	303	95.7	21	4.3	<0.001
65–74	291	90.0	36	10.0	
≥75	167	80.0	28	20.0	
Gender					
Men	335	93.8	28	6.2	0.020
Women	426	88.6	57	11.4	
Residential area					
Urban	582	91.5	57	8.5	0.426
Rural	179	89.3	28	10.7	
Educational level					
Below elementary school	313	84.9	54	15.1	<0.001
Middle school	148	92.5	15	7.5	
High school	177	94.5	10	5.5	
College	123	96.9	6	3.1	
Spouse, yes	581	92.6	53	7.4	0.002
Job, yes	347	89.4	32	10.6	0.176
Household income					
Low	289	90.8	38	9.2	0.850
Low-middle	197	91.8	17	8.2	
Middle-high	153	89.4	19	10.6	
High	122	92.3	11	7.7	
Current smoking, yes	125	91.5	15	8.5	0.813
Monthly drinking, yes	344	94.0.	28	6.0	0.004
Comorbid conditions					
0	328	92.9	30	7.1	0.011
1	267	92.2	26	7.8	
≥2	166	85.1	29	14.9	
Time since HTN diagnosis (years)	9.76 ± 8.36		9.57 ± 7.86		
≤5	293	92.1	32	7.9	0.674
6–10	212	89.8	27	10.2	
≥11	256	90.5	26	9.5	
Daily medication, yes	715	90.7	79	9.3	0.315
BMI (kg/m^2^)	25.10 ± 3.20		24.63 ± 3.30		
<18.5	197	87.3	29	12.7	0.150
18.5–22.9	101	93.8	8	6.2	
23–24.9	87	88.5	11	11.5	
≥25	376	92.8	37	7.2	
Systolic BP (mmHg)	128.02 ± 15.76		126.04 ± 15.24		0.259
Diastolic BP(mmHg)	75.4 4 ± 10.40		73.82 ± 9.87		0.157

Notes: BMI = body mass index; BP = blood pressure; HTN = hypertension; *n* = unweighted sample size; *N* = weighted sample size. ^a^ *p* values were obtained by the Rao-Scott χ^2^ test.

**Table 2:** Comparison of physical activity and sleep duration among hypertension patients with and without depressive symptoms (*n* = 846).

Variables	Without Depressive Symptoms		With Depressive Symptoms		*p*-Value ^a^
	(*n* = 761, *N* = 3,835,776)		(*n* = 85, *N* = 379,127)		
	*n* or Mean ± SD	Weighted %	*n* or Mean ± SD	Weighted %	
Physical activity (MET-min/week)	1347.27 ± 3941.09		491.41 ± 1116.89		
Low PA	345	84.2	71	15.8	<0.001
Moderate to vigorous PA	416	96.9	14	3.1	
Sleep duration (h/night)	6.70 ± 1.49		6.36 ± 2.08		
Short (<7)	323	89.2	42	10.8	<0.001
Normal (7–8)	387	95.4	20	4.6	
Long (≥9)	51	73.6	23	26.4	

Notes: *n* = unweighted sample size; *N* = weighted sample size; MET = total metabolic equivalents; PA = physical activity. ^a^ *p* values were obtained by the Rao-Scott χ^2^ test.

**Table 3:** Predictors of depressive symptoms in patients with hypertension (*n* = 846).

Predictors	Depressive Symptoms			
	Unadjusted		Adjusted	
	OR (95% CI)	*p*-Value ^a^	OR (95% CI)	*p*-Value ^a^
Age (Ref. 24–64 years)				
65–74	5.22 (2.57–10.59)	<0.001	1.84 (0.93–3.64)	0.079
≥75	2.37 (1.19–4.72)	<0.001	3.15 (1.37–7.24)	0.007
Gender (Ref. Male)				
Female	1.96 (1.11–3.48)	0.021	0.96 (0.42–2.19)	0.923
Educational level (Ref. College school)				
Below elementary school	5.62 (2.21–14.28)	<0.001	3.06 (1.01–9.29)	0.048
Middle school	2.57 (0.89–7.36)	0.078	2.48 (0.82–7.43)	0.106
High school	1.83 (0.57–5.94)	0.311	1.99 (0.63–6.39)	0.241
Spouse (Ref. No)				
Yes	0.48 (0.29–0.77)	0.003	0.76 (0.41–1.41)	0.382
Monthly drinking (Ref. No)				
Yes	0.46 (0.27–0.79)	0.005	0.79 (0.42–1.49)	0.454
Comorbid conditions (Ref. 0)				
1	1.11 (0.61–2.04)	0.730	0.91 (0.45–1.84)	0.797
≥2	2.29 (1.28–4.09)	0.005	1.50 (0.72–3.12)	0.275
Physical activity (Ref. Moderate to vigorous PA)				
Low PA	5.93 (3.06–11.52)	<0.001	4.42 (2.19–8.89)	<0.001
Sleep duration (Ref. Normal: 7–8 h/night)				
Short (≤6)	2.52 (1.39–4.55)	0.003	2.18 (1.11–4.28)	0.024
Long (≥9)	7.45 (3.49–15.93)	<0.001	4.09 (1.83–9.13)	0.001

Notes: CI = confidence interval; PA = physical activity; *n* = unweighted sample size; OR = odds ratio. ^a^ *p* values were obtained by complex weighted sample, Adjusted Model Nagelkerke R^2^ = 0.218.
